# Dao Yin (a.k.a. Qigong): Origin, Development, Potential Mechanisms, and Clinical Applications

**DOI:** 10.1155/2019/3705120

**Published:** 2019-10-21

**Authors:** Xiaorong Chen, Jiabao Cui, Ru Li, Richard Norton, Joel Park, Jian Kong, Albert Yeung

**Affiliations:** ^1^Faculty of Physical Education, Shenzhen University, Shenzhen 518060, China; ^2^South China Normal University, Guangzhou 510006, China; ^3^Depression Clinical and Research Program, Massachusetts General Hospital, Boston, MA, USA; ^4^Department of Psychiatry, Massachusetts General Hospital and Harvard Medical School, Boston, MA, USA

## Abstract

Dao Yin is a form of exercise combining physical movements, mental focus, and breathing originated in ancient China. In this review, we introduce the history in the development and the scope of Dao Yin, the relationship between Dao Yin with Taoist culture and Qigong, and the potential mechanisms of how Dao Yin promotes health and alleviate illnesses. Empirical research studies using Dao Yin for treatment of lumbar spondylosis, peripheral musculoskeletal diseases, cervical spondylosis, heart diseases, central nervous system disorders, immunological dysfunction, and psychological disorders are detailed.

## 1. Introduction

Chinese Dao Yin (导引) exercise has been widely applied to medical treatment and health preservation since ancient times, and it is also acknowledged as an effective traditional orthopaedic therapy by both ancient and modern traditional Chinese medicine. “Dao” (导) means to guide *qi*, the internal vital energy of the body, so as to create an internal balance, and “Yin” (引) means to stretch the body so as to gain strength and flexibility [1]. Through physical movements, practicing Dao Yin exercise can create the harmony of *qi* and blood in the body, thus achieving the purpose of preventing disease, improving health and accelerating the recovery of limb function. Dao Yin is a kind of body exercise that involves breathing, massaging through stretching and twisting arms and legs, and mental focus which act together to direct the flow of *qi* [2–5].

Dao Yin exercise has a broad and a narrow meaning. In the broad sense, any active Chinese exercise theory or method that aims to achieve the “harmony between the internal direction of vital energy and the external stretching of the body” can be considered as a form of “Dao Yin exercise.” This can be practiced through Tu Na (expiration and inspiration), body stretching, massaging, Lian Dan (inner alchemy), Zuo Chan (sitting in meditation), inward contemplation, and so on. These methods are habitually categorized into “Dao Yin” and “Xing Qi (promoting the circulation of *qi*)” in line with the traditional standards in ancient times. Both “Dao Yin” and “Xing Qi” take the cultivation of essence (the fundamental substance which maintains the functioning of the body), *qi,* and spirits as their core, but they require different proportions of these three elements. Laying a foundation for the so-called “static exercise” developed by later generations, Xing Qi is mainly based on breathing exercises aided by body and mental training. In contrast, as the forerunner of the so-called “dynamic exercise” developed by later generations, Dao Yin centers on body exercise supplemented by breathing and mental training, which is considered as “Dao Yin exercise” in its narrow sense. The objective of this research, especially in its discussion of therapeutic effects of Dao Yin exercise, refers to Dao Yin exercise in its narrow sense, that is, “dynamic exercise” mainly based on body movements aided by breathing and mental adjustments. Thus, Dao Yin exercise hereinafter can be extended to traditional Qi Gong (气功), health Qi Gong, and traditional martial arts represented by Tai Ji Quan with health-preservation effects and so on.

## 2. Origin of Dao Yin Exercise

As the most indigenous health-preservation method in China, Dao Yin exercise can be traced back to ancient times (about 2146 BC). In that era, as recorded in *Lü*'*s Spring and Autumn Annals · The Old Tunes*, “when the tribe of Tao Tang began to govern the world, Yin (here “Yin” refers to the opposite of Yang) was blocked and stagnant, the water courses were silted and there was a huge flood. As a result, people were depressed and their bones and muscles began to atrophy. Therefore, grand dance was created to reinvigorate the bones and muscles of people.” In other words, a kind of dance was created to let patients move their limbs so as to promote the flow of their *qi* and blood as well as to reinvigorate their bones and muscles, which is exactly the original form of “Dao Yin.” The term “Dao Yin” was first used in Zhuangzi: *Chapter 15 Rigid and Arrogant* [6]—“Huffing, puffing; grunting and groaning; expelling the old breath and taking in the new; undertaking physical exercise like a bear or a bird to preserve the body and soul; long life his sole concern: this is what is approved, this is the Tao of the scholar who infuses his self with breath, feeding his body, hoping to live as long as Peng Zu.” It is believed that long life can be achieved through physical exercise by imitating the limb movements of animals, such as a bear or a bird, meanwhile with the aid of Tu Na. Compared with general physical activities, Dao Yin has its special features. As stated in Xuan Jian Dao Yin exercise [7], which is the 36^th^ volume of *Yun Ji Qi Qian*, an anthology of the *Taoist Canon*”, “Dao Yin must follow the principle of peace; head must be raised slowly and the body must be stretched rhythmically. That is to say, when practicing Dao Yin, one is supposed to keep himself peaceful, and all the physical movements should be performed with a moderate rhythm [1].

During the Qin and Han Dynasties (221 BC–220 AD), the continuous development of traditional Chinese medicine brings about further progress in Dao Yin exercise. According to *Synopsis of Prescriptions of the Golden Chamber* by Zhang [8], “one should carefully protect one's body resistance and avoid the attack of climatic pathogenic factors. Otherwise, channels and collaterals will be violated and health endangered. In case pathogenic factors have invaded the channels and collaterals, medical treatment should be given in time to stop the transmission of pathogenic factors into the viscera and bowels. If there is heaviness and uneasiness in the extremities, Dao Yin, Tu Na, acupuncture, and ointment rubbing should be practiced to clear the nine orifices.” [8] Here, Dao Yin is juxtaposed with Tu Na, which coincides exactly with the advocation of “huffing, puffing; grunting and groaning; expelling the old breath and taking in the new; undertaking physical exercises like a bear or a bird to preserve the body and soul” in *Zhuangzi: Chapter 15 Rigid and Arrogant* [6], suggesting that Dao Yin should be practiced together with breathing exercise and can yield significant influence on man's channels and collaterals. Furthermore, it is also pointed out in *Central Treasury Canon* [9] by Hua (a legendary doctor at the end of the Han Dynasty), “practicing Dao Yin could drive out pathogenic factors in one's joints”; “if one does not practice Dao Yin when he or she should, pathogenic factors will invade his or her joints, resulting in incurable pain.” Ancient Chinese doctors in the Han Dynasty began to have a more comprehensive understanding of therapeutic effects of Dao Yin exercise on diseases, which lead to a broader range of its application to the medical field.

The Sui and Tang Dynasties (581 AD–907 AD) witnessed the advent of the four classics of traditional Chinese medicine, among which *General Treaties on Causes and Manifestations of All Diseases* has the greatest influence on later generations. After sorting out and categorizing the Dao Yin methods recorded in this book, Dai Jin-gang in his doctoral dissertation has named these Dao Yin methods, concluded their functions, explained their movements, and elucidated their principles. According to his research results, *General Treaties on Causes and Manifestations of All Diseases* consists of a total of 50 volumes, expounding on 1739 syndromes, which fall into 71 types of internal medicine, surgery, gynaecology, and paediatrics, providing all together 287 Dao Yin methods for specific treatment of some syndromes. In this book, the health-preservation Dao Yin therapies for the treatment of different syndromes can be regarded as the earliest exercise prescription in the world [10].

In summary, Dao Yin exercise has prevailed in the traditional Chinese medical field for thousands of years. According to related records in ancient books and literature, Dao Yin exercise is a unique system of health-preservation and disease-treatment methods with specific contents and functions, cultivated in the profound and time-honoured cultural soil of the Chinese nation [1]. Different from general physical exercise, Dao Yin exercise integrates the body with breathing, mental focus, and emotional regulation; it is not strenuous but requires the control of rhythm and temperament, which aims to promote health, prevent disease, and treat illness by actively practicing this exercise.

## 3. Relations of Dao Yin Exercise with the Taoist Culture and Qi Gong

### 3.1. Dao Yin Exercise and Taoist Culture

As defined by *Ci Yuan* (the earliest modern encyclopaedic Chinese phrase dictionary), “Dao Yin” refers to “a Taoist health care technique, which aims to enrich *qi* and blood as well as relieve the body by doing breathing exercise, raising the body up and stooping down, and bending and stretching limbs.” [11]. In *Ci Hai* (a large-scale dictionary and encyclopaedia of Standard Mandarin Chinese), “Dao Yin” is defined as “an ancient Chinese method for health promotion and disease treatment,” which is also referred to as “Taoist Yin” [12]. It is thus clear that Dao Yin exercise has a close connection with the Taoist culture. From a Taoist point of view, Tao is everything (“From Tao there comes one. From one there comes two. From two there comes three. From three there comes everything” [13]), and everything is taken as a whole characterized by interconnection, interdependence, and constant change. This Taoist philosophy has a direct impact on the theoretical framework of *Inner Canon of the Yellow Emperor* [14] (one of the four classics of traditional Chinese medicine as mentioned hereinabove), manifested by its statement “as the heaven has sun and moon, man has two eyes; as the earth has nine regions (according to ancient Chinese territory division), man has nine orifices; as a year has four seasons, man has four limbs; as music has five notes (according to traditional Chinese musicology), man has the five internal organs (heart, liver, spleen, lungs and kidneys); as the heaven has six terms, man has the six hollow organs (gallbladder, stomach, large intestine, small intestine, bladder, and San Jiao),” “as the earth has twelve major rivers (according to ancient Chinese geography), man has twelve conduit vessels (passages through which vital energy circulates).” These descriptions in *Inner Canon of the Yellow Emperor* demonstrate the close connection between the macrocosm and the microcosm in the Taoist culture. Based on the Taoist cosmology, theories of the two *qi* (Yin and Yang) and the five agents (metal, wood, water, fire, and earth) were further developed, and an entire system of religious doctrines, theories, and methodologies were also established on the basis of Taoist concepts such as “Tao,” “Tai Ji” (great ultimate), “Liang Yi” (the two primary forms, i.e., Yin and Yang), and “Ba Gua” (the eight symbols in the Taoist cosmology) [14]. A series of Taoist health-preservation theories, including “quiet nonaction,” “returning to nature,” “conforming to nature,” “valuing man and life,” “unity of man and nature,” “correspondence between man and nature,” “life controlled by oneself” and “health preservation in line with Tao” [15], have laid a relatively complete theoretical foundation for the development of Dao Yin health-preservation exercise.

### 3.2. Dao Yin Exercise and Qi Gong

Despite its long existence since ancient times, it is not until modern times that the term Qi Gong (Gong, or “Kung,” is often translated as cultivation, work, or exercise) has been widely used to refer to the self-exercise method, which is also a complex system, that aims to improve the body and mind through the utilization of consciousness. In ancient times, there were no less than 30 names for Qi Gong, such as Tu Na, Xing Qi, Bu Qi (infusing *qi*), Fu Qi (absorbing *qi*), Dao Yin, Lian Dan, Xiu Tao (cultivating oneself according to the Taoist doctrines), and Zuo Chan. Actually, Qi Gong has many forms, and those many forms called by the various names hereinabove are different subtypes of Qi Gong and can be further categorized into Massage Gong, Dao Yin Gong, and Health-Preservation Gong, according to their different effects on human body. In the 1950s, due to the vigorous promotion of the famous Chinese Qi Gong expert Liu Gui-zhen, the term Qi Gong was officially acknowledged by the government [16].

In recent times, some scholars pointed out that the congruence of “Qi Gong” and “Dao Yin exercise.” For example, as Guo pointed out in his *Slavery Age*, “what ancient people called Tao (or Dao) Yin is exactly what we call Qi Gong now [17].” Qu [18], professor at Beijing University of Chinese Medicine, also holds the same view, arguing that health Qi Gong is the modern version of Dao Yin exercise. In her doctoral dissertation based on a scrutiny of literature and a rigorous logical analysis, Wei [19] concludes that “Dao Yin exercise, Qi Gong, and health Qi Gong are cultural phenomena of health preservation in different times, andhealth Qi Gong has been a new form of Dao Yin regimen since China's reform and opening up and meanwhile a carrier of traditional Chinese culture.”

## 4. Modern and Contemporary Development of Dao Yin Exercise

The period from the Opium War in 1840 to the founding of the People's Republic of China in 1949 witnessed a slow down in the development of Chinese Dao Yin exercise, but some achievements were still made. In 1858, the doctor and government official Pan Wei finished *Arts for Health*, a monograph on Qi Gong which advocates that, in the face of disease, “prevention” is more important than “treatment,” and that Qi Gong is the method for the former [20]. In 1881, *Arts for Health* was facsimiled and illustrated by Wang The new edition was entitled *Illustrated Handbook on Nei Gong* (inner exercise), which elaborates on the practice of dynamic exercise, explains some specific methods, and provides corresponding illustrations [21]. Wu Shang-xian, an expert in external treatment, and Liu and Zhang, who mastered both traditional Chinese and Western medicine, also made certain contributions to the study of Qi Gong during this period of time [22]. Since the early years of the Republic of China (1912–1949), Jing Zuo (sitting in silence) was popular among the intellectual class. Jiang Wei-qiao's *Jing Zuo Handbook by Yin Shi Zi* (“Yin Shi Zi” is the Taoist monastic name of Jiang Wei-qiao) is a masterpiece on sitting in silence. It was also during this period of time that China's Qi Gong began to spread in Europe [23].

In 1974, China unearthed *Pictures of Dao Yin Exercise* from the Mawangdui Tombs, finding out the earliest Chinese health-keeping exercise illustrated by 44 pictures of men and women practicing gestures of Dao Yin. Each illustration has a title, which is the name of the corresponding gesture, followed by an explanatory note which often mentions the therapeutic effect on disease. The Dao Yin exercise illustrated by these pictures basically falls into four forms, namely, breathing exercise, limb exercise, exercise with equipment, and therapeutic exercise. These all demonstrate how Chinese people practiced exercise to promote health more than two thousand years ago.

In 1984, over a thousand bamboo and wooden slips were excavated from the Western Han Dynasty Tombs in Zhangjiashan, Jiangling, Hubei Province. Among them, there are two medical documents: *On Conduit Vessels* and *On Yin*. *On Conduit Vessels* was written in clerical script (an ancient style of calligraphy current in the Han Dynasty) in three parts: part one talks about how to promote the circulation of *qi* (Xing Qi) and regulate daily life in response to different seasons; part two introduces gestures and movements of Dao Yin; part three discusses how Dao Yin and Xing Qi are connected with health preservation and disease treatment [24]. According to *On Yin*, Dao Yin exercise has both repetitive movements, which are easy to practice, and serial movements, which requires the performance of various gestures in succession. *On Yin* also concludes the beneficial effects of Dao Yin exercise on 24 parts of the human body [25].

Under the organization and promotion of the Health Qi Gong Management Centre of China's General Administration of Sport, which was established in June 2001, four forms of Qi Gong, namely, Yi Jin Jing (muscle change classic), Wu Qin Xi (five-animal exercise), Liu Zi Jue (six healing sounds), and Ba Duan Jin (eight silken movements) have been recreated and have gained worldwide popularity [23].

In order to adapt more forms of excellent traditional health Qi Gong into versions that are suitable for common people's daily exercise, the Health Qi Gong Management Centre of China's General Administration of Sport started to create new forms of health Qi Gong in 2007. Strictly in the form of academic study, five forms of health Qi Gong, namely, Tai Ji Yang Sheng Zhang (Tai Ji Stick Health Preservation Exercise), Shi Er Duan Jin (twelve silken movements), Dao Yin Health Preservation 12 Methods, Mawangdui Dao Yin Exercise, and Grand Dance, were eventually created [26].

## 5. Potential Mechanism of Dao Yin Exercise on the Body

### 5.1. Potential Mechanisms from a Traditional Chinese Medicine Perspective

Traditional Chinese medicine has a close connection with and laid a solid theoretical foundation for Dao Yin exercise, whose principles of health preservation and disease treatment are mainly based on theories of traditional Chinese medicine, those being on “Ying and Yang,” “*qi*,” “main and collateral channels,” “essence, *qi*, and spirits,” “nature-life,” “*qi*, blood, and body fluid,” etc. Ru holds that practicing Dao Yin exercise will enhance the flexibility of limbs and stretch the movements of the body, thus dredging the inner passages through which *qi* and blood circulate so as to promote the circulation of *qi*. In this way, *qi* is guided through these passages and inner harmony is created, eventually reaching a healthy state characterized by strong muscles, bones, flexible joints, and smooth circulation of *qi* and blood [5]. Based on scientific research, Qu and Zheng believed that stretching and relaxing muscles along the regular meridians by physical exercise will clear conduit vessels in the whole body and make the circulation of *qi* and blood smooth. Free circulation will dispel pains, which finally leads to improvement in movement functions [2].

Zhang Guang-de, a famous Chinese Dao Yin expert, categorized principles of health preservation and disease treatment by differentiating Dao Yin exercise into the “regulation of breathing” and the “regulation of the body.” The “regulation of breathing” Dao Yin exercise emphasizes the close coordination between movements and abdominal breathing that is soft, even, deep, and long. Abdominal breathing with such characteristics can increase the range of contraction and relaxation of the diaphragm, bringing about benefits such as a massaging of the liver, stomach, spleen, and intestine, to increase circulation of blood and strengthen the ligaments between the smooth muscles of organs. During this type of breathing, due to the wide range of diaphragm contraction and relaxation, the diaphragm can be exercised and strengthened, thus deepening as well as slowing down the breathing and leading to the state of breathing economy. In this way, the lungs can get a longer rest after each breath, which not only helps to inhale more fresh air but also makes the lungs and respiratory muscles less prone to fatigue, thereby making the ventilatory function thus improved.

The “regulation of the body” Dao Yin exercise has specific parameters: firstly, “every movement involves spins.” During spinning movements, the area of force applied to the spinned part can be expanded, and channels and collaterals in the corresponding part can be stimulated. Secondly, “relaxation is combined with tension and dominates the whole process.” Mental relaxation can let the brain rest, dispel all distracting thoughts, and help the limbs relax; limb relaxation can increase the number of open muscle capillaries, relax the blood vessel walls, accelerate blood circulation, and change the functional state of the vasomotor centre. Thirdly, “joints, fingers, and toes should be exercised.” Rhythmically moving the joints of the elbows and the lower part of the knees, the fingers and the toes can promote the circulation of *qi* and blood in the conduit vessels so as to achieve lateral balance and treat diseases. Fourthly, “one should maintain soft, slow, coherent, and flexible.” When practicing Dao Yin exercise, one should keep the movements soft, slow, coherent, and elastic rather than stiff, slack, inflexible, and disjointed. Such movements can clear channels and collaterals, regulate as well as nourish *qi* and blood, improve functions of the five internal organs, eliminate food stagnation and blood stasis, and finally achieve the effects of disease treatment when one is sick as well as health preservation when one is well [27].

### 5.2. Potential Mechanisms from Neuropsychological and Immunological Perspectives

**Figure 1:**
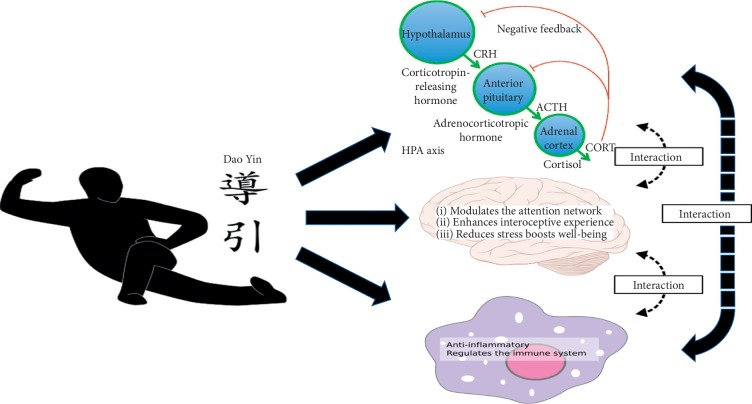
Diagram of the interaction of Dao Yin exercise with inflammatory markers and particular brain mechanisms.

Studies of mind-body interventions may shed light on the mechanisms of Dao Yin's effect on the body. Though various forms and styles of Dao Yin have developed throughout the years, we believe that Dao Yin may modulate health through three key mechanisms (Figure 1). The first mechanism works through the brain, i.e., enhancing interoception, regulating the cognitive control network, and modulating emotion processing to boost well-being [28, 29]. In support of this hypothesis, studies have found a modulation effect of meditation on the structure and function of the anterior insula and ACC [27–32], key regions involved in interoception. In addition, studies have shown that Tai Chi and Baduanjin, two traditional Dao Yin methods, can significantly modulate the cognitive control network in both healthy subjects [33] and chronic pain patients [34], as well as the default mode network, a network associated with self-inferencing in older adults [35].

The second mechanism is through the attenuation of hypothalamus-pituitary-adrenal (HPA) axis reaction to stress. Under stress, the amygdala is excited, which leads to the activation of the hypothalamus, which triggers the pituitary gland to secret the adrenocorticotropic hormone (ACTH). The ACTH stimulates the adrenal cortex to produce cortisol, which increases blood pressure and blood sugar levels, turns fatty acids into energy, and suppresses the immune system. At the same time, stress triggers the sympathetic nervous system to stimulate the adrenal medulla to produce catecholamine hormones, such as adrenaline (epinephrine) or noradrenaline (norepinephrine). This pathway prepares the body for fight or flight actions. The sympathetic nervous system leads to pupillary dilation, increased heart rate and blood pressure, bronchial dilatation, and decreased movement of the large intestine. We believe that Dao Yin counteracts many of the stress responses, presumably by activating the parasympathetic nervous system, and leads to the “relaxation response” [36].

Thirdly, Dao Yin may also modulate the immune system, enhancing anti-inflammation. Recent studies have suggested that traditional Dao Yin methods such as Tai Chi may reduce stress and modulate the inflammatory process [37, 38]. For instance, Jin [39] found that the practice of Tai Chi can decrease salivary cortisol concentrations. Compared to baseline levels, subjects reported less tension, depression, anger, fatigue, confusion, and state anxiety and had also felt more vigorous. Irwin and Olmstead [40] evaluated the effects of Tai Chi on circulating markers of inflammation in older adults and found that, among those with high IL-6 levels at entry, Tai Chi produced a drop in IL-6 levels to levels comparable to those found in Tai Chi and health education subgroups with low IL-6 levels at entry. Meanwhile, IL-6 in the health education (HE) subgroups remained higher than the Tai Chi subgroup with low IL-6 at entry. Decreases in depressive symptoms in the two treatment groups correlated with decreases in IL-6.

It is worth noting that the three mechanisms may not function independently but rather interact with each other. For instance, literature suggests that long-term stress may initiate cognitive, affective, and possibly biological processes that increase one's risk for depression and other disorders [41, 42], and inflammation may play an important role in this process. Specifically, neuroinflammatory sensitization provoked by stress elicits profound changes in human body [41, 42]. As mentioned above, Dao Yin may reduce stress by focusing on the internal status of the human body (interoception) and enhancing well-being. The hypothalamus, anterior insula, and ACC may be involved in this process [41]. Thus, the three mechanisms may work together to improve and maintain health.

## 6. Therapeutic Effects of Dao Yin Exercise

Dao Yin exercise is a series of exercises that are very suitable for the health preservation of middle-aged and elderly people. It is not only an aerobic exercise but also a treatment method for a variety of diseases. According to the categorization of experts in this field [1], the practice of Dao Yin exercise mainly falls into four forms: mental activities, breathing regulation, limb movements, and self-massage. As a traditional exercise therapy, Dao Yin exercise integrates the above four exercises into a whole.

The medical value and therapeutic effects of Dao Yin exercise on various diseases have been fully recognized by doctors for many generations. With constant enrichment, development, and improvement, the combination of Dao Yin and modern exercise therapies has been explored in clinical research and applied to the prevention and intervention of physical and mental illnesses. Existing research into the therapeutic effects of Dao Yin exercise on physical illnesses mainly focus on lumbar spondylosis, limb diseases, cervical diseases, heart disease, central nervous disorders, immune dysfunction, and so on [7]. As for its intervention as treatment of limb joints, lumbar vertebrae, cervical vertebrae, mental illness, and the physique, the research subjects tend to be middle-aged and elderly people, while the research results are quite fruitive. As a great amount of research has proved the effectiveness of Dao Yin exercise, its philosophy, forms, methods, and therapeutic effects have gradually gained people's recognition.

### 6.1. Therapeutic Effects of Dao Yin Exercise on Lumbar Spondylosis

Lumbar spondylosis is mostly caused by acute or chronic injuries of the spinal column or its surrounding soft tissues, the degeneration of the lumbar intervertebral disc, lumbar hyperosteogeny, etc. [43]. Clinically, it is mainly manifested by low back pain, lumbar motor disturbance, and pain in waist and lower extremities. Excessive sitting causes the imbalanced strength of the lumbar joints and muscles; that is, the tense muscles are always tense, while the relaxed muscles are always relaxed, resulting in poor blood circulation and decreased metabolic function. Dao Yin exercise generally involves wide stretches to exercise and relax muscles. As proved by a wealth of research results, practicing Dao Yin exercise does have therapeutic effects on the rehabilitation of lumbar spondylosis and the enhancement of lumbar and abdominal strength.

According to Qu and Zheng [2], practicing Dao Yin exercise, through its strengthening of muscles of the back, enhances muscle strength and endurance, provides stable support for the lumbar vertebrae, stimulates the nerves, and thereby maintains the stability of the spinal column and prevents low back pain.

Bai et al. [44] believed that the Wu Qin Xi, or five-animal exercise, can effectively stretch the spinal column in all directions. This is done so that the multifidus muscle can be effectively exercised, thereby strengthening the function of the multifidus muscle, increasing the stability of the spinal column and alleviating low back pain.

The research conducted by Ding and Wang [45] shows that practicing Ba Duan Jin, or eight silken movements, enhances the strength of the back and abdominal muscles, thereby significantly improving lumbar lordosis and sacral inclination. In other words, practicing Ba Duan Jin effectively controls the movement of the spinal column and pelvis and maintains their stability, thereby relieving low back pain and promoting lumbar activity.

Li et al. [46] randomly divided the middle-aged and elderly patients with chronic low back pain into two groups. The experimental group was treated by letting them practice standing-position Ba Duan Jin for 12 weeks, while the control group was treated with painkillers. Visual analogue scale (VAS), Oswestry dysfunction index, lumbar lordosis, sacral inclination, and lumbar activity of the patients in the two groups before and after the treatment were compared. The results showed that, before the treatment, there was no difference between the two groups in all the indicators, and that after the treatment, all the indicators of the two groups were improved, with more significant improvement found in the experimental group (*P* < 0.05 or *P* < 0.01). Therefore, it is concluded that standing-position Ba Duan Jin exercise can improve lumbar lordosis and sacral inclination, thereby significantly relieving chronic low back pain and improving lumbar activity.

Chen et al. [47] sifted out 60 patients with chronic low back pain from cases that met the selection criteria. These patients were asked to practice lumbar Dao Yin exercise, and the clinical effects were assessed by VAS and the JOA (Japanese Orthopaedic Association) scales for spinal cord function. After assessing the spinal cord function of the patients, it was found that practicing lumbar Dao Yin exercise had let the back muscles fully relax and stretch, which helped eliminate the fatigue of the back muscles and trained the muscles with load. This effect contributed to the enhancement of muscle strength and endurance as well as the improvement of lumbar function.

### 6.2. Therapeutic Effects of Dao Yin Exercise on Peripheral Musculoskeletal Diseases

People with musculoskeletal diseases often do not want to move the area where pain is located. The lack of limb motions leads to decreased activity and flexibility of the joints and even the possibility of inflammation [48]. Dao Yin exercise is practiced slowly at a constant speed, mainly involving the movements of the limbs and requiring a certain amount of exercise. Due to its benefits in physical coordination improvement [49] and the balance between the limb muscles and muscle groups [49], Dao Yin exercise can be well applied to the treatment of peripheral musculoskeletal diseases with therapeutic effects.

In the article “Characteristics of Traditional Dao Yin Limb Exercise from the Angle of Joint Motions” by You and Wang [50], the movement characteristics of Dao Yin exercise is concluded as the stretching training of the limbs and the isometric contraction of the opposing muscle groups.

According to Ru [5], a Dao Yin exerciser stretches the body under the guidance of his or her active consciousness. Such activity stretching the body not only balances the muscle contraction force around the joints of the limbs but also increases the volume of the limb tissue fluid, thus forming a stretch-fluid loop. Such exercise, if practiced for a long term, also brings about benefits to the central nervous system's ability to regulate and control the muscles, thereby enhancing limb flexibility and physical agility.

Chen et al. [51] recruited 80 patients with knee arthritis from those who met the selection criteria (aged 40–75) and randomized them into the control group and the experimental group; all the patients were treated with the oral administration of “Yangxue Ruanjian Capsule,” Chinese herbal fumigation and washing. The experimental group additionally practiced a self-created set of Dao Yin exercises, including the forms of Pushing Eight Horses Forward, Up-and-Down Stance, Standing Stance, and Horse Stance. The JOA Functional Assessment Scale of Knee Joints and VAS were used to evaluate the effects, and the results of the two groups obtained by statistical comparison were significantly different (*P* < 0.01). It is thus concluded that the addition of Dao Yin exercise to conventional basic treatment has significant therapeutic effects on knee arthritis.

In two more recent studies [52, 53], Liu and colleagues compared the effect of Tai Chi, Baduanjin, and cycling in patients with knee OA and found (1) all three exercises can relieve pain symptoms and increase serum programmed cell death protein 1 (PD-1) levels and (2) different exercises can modulate both common and unique brain pathways.

### 6.3. Therapeutic Effects of Dao Yin Exercise on Cervical Spondylosis

There is evidence that cervical spondylosis is becoming an increasingly common disease among more and more young people, and that, in the next 50 years, it will be become a clinically important disease similar to low back pain. People who work at the desk for long hours have their neck muscles in a long-term state of tension without enough relaxation, resulting in decreased elasticity of the muscles and thereby leading to adhesion. Tissue adhesion irritates and oppresses the nerves of the spot, which causes pain. The slow-paced stretching of Dao Yin exercise promotes blood circulation, increases blood supply to the neck, and brings about benefits to nutrition and recovery. There are certain research results, indicating the clinical efficacy of Dao Yin exercise on cervical spondylosis.

In the controlled trials conducted by Jing et al. [54], 120 patients who were diagnosed with cervical spondylosis and met the trial inclusion criteria were taken as research subjects and randomly divided into the control and the experimental groups, each consisting of 60 patients. Both groups were treated with spinal manipulation in a lying position, while the experimental group additionally received Dao Yin therapy. The results showed that there was significant statistical difference before and after the treatment of each group and between the two groups after the treatment; compared with pure spinal manipulation, its combination with Dao Yin exercise yielded more significant clinical therapeutic effects on cervical spondylosis.

By conducting a prospective cohort study, Zhu et al. [55] researched into the prevention and treatment of cervical spondylosis by cervical-vertebra Dao Yin exercise and health education. The so-called cervical-vertebra Dao Yin exercise refers to the movements of leftward-rightward and forward-backward stretching of the cervical vertebra, chest expanding, and body stretching against the cervical vertebra. Healthy software professionals were selected as the research subjects. Those in the intervention group practiced cervical-vertebra Dao Yin exercise and received health education, while the control group received no intervention. Over the course of 3 years, the incidence rates were observed in both groups, with a statistically significant difference in the incidence rates between the two groups (*P* < 0.01); the risk of cervical spondylosis in the control group was significantly higher than that in the intervention group.

By using the random number table, Wang et al. [56] randomly divided 60 patients with cervical spondylosis, who were treated in hospital, into the treatment group and the control group, each consisting of 30 patients. Those in the control group were treated with manipulation therapy once every other day, that is, 3 times a week, for three consecutive weeks. In addition to the treatment in the control group, those in the treatment group were further treated with cervical-vertebra Dao Yin exercise twice every day (once in the morning and once in the evening), 5 days a week for three consecutive weeks. Significant improvement was found in the treatment group compared with the control group (*P* < 0.05).

### 6.4. Therapeutic Effects of Dao Yin Exercise on Heart Diseases

Practicing Dao Yin exercise also has positive effects on improving the cardiovascular and immune functions of middle-aged and elderly women. Based on a scrutiny of literature, it is found that different forms of Dao Yin exercise produce different curative effects on heart disease. For example, practicing Liu Zi Jue strengthens the internal functions of the human body, and in the process of breathing exercise, the potential ability of the viscera can be fully triggered and mobilized to resist the invasion of illness and prevent cardiovascular and cerebrovascular disease that may occur with the increase of people's age [57, 58]. With the slowness and continuity of its movements, practicing Wu Qin Xi not only increases blood volume of venous return but also strengthens myocardial contractility to a certain degree, thereby enhancing the pumping force of the heart in the long run [59]. As both traditional Chinese and Western medicine attaches great importance to kinesitherapy in cardiac rehabilitation, existing research demonstrates that Dao Yin exercise, as a specific form of kinesitherapy in traditional Chinese medicine, produces significant benefits to the rehabilitation of heart disease.

In the article “Clinical Application of Strengthening Yang Guidance in the Cardiac Rehabilitation” by Li et al. [3], it is believed that Dao Yin exercise (referred to as “Guidance” by Li et al.) emphasizes the integration of the dynamic and the static which aims to regulate one's body, breathing, and mind. These “three regulations” will contribute to the balance of autonomic nerves, relieve the stress of sympathetic nerves, and allow the heart rate, cardiac output, and blood pressure to have a modest adjustment, thus yielding positive effects on the prevention and adjuvant treatment of cardiovascular disease. It was proposed that long-term practice of this method could elevate the spirits and emotions of a patient, reduce mental stress, and promote emotional stability.

Xi and Wang [60] took 80 middle-aged and elderly women as the research subjects, who were randomly divided into the exercise group (*n* = 40, 2 dropout cases) and the control group (*n* = 40 patients); the patients in the exercise group practiced exercise together 3 times a week, one hour each time, for a total of 20 weeks, while the control group did no physical exercise. Before and after the 20-week experiment, each subject's cardiovascular function indicators, IgA, IgG, and IgM were tested, respectively. The test results suggested that, after 20 weeks of exercise, the exercise group showed different degrees of decrease in heart rate (HR), pulse pressure (PP), systolic pressure (SP), diastolic pressure (DP), significant increase (*P* < 0.05) in stroke volume (SV), cardiac output (CO), stroke index (SI), and cardiac index (CI), and nonsignificant increase (*P* > 0.05) in the IgM level. After 20 weeks of experiment, the control group showed no significant difference (*P* > 0.05) in all indicators.

### 6.5. Therapeutic Effects of Dao Yin Exercise on Central Nervous System Disorders

The central nervous system, which consists of the brain and the spinal cord, serves as the major part of the human nervous system. Information from all parts of the body is received, integrated, and processed by the central nervous system into different coordinated activities or stored in the central nervous system to be the neural basis of learning and memory [61]. The treatment of motor dysfunction caused by central nervous cellular necrosis should firstly focus on the functional recovery of central nervous cells. For instance, foot drop in the cases of stroke and cerebral palsy happens due to tibialis anterior palsy and plantar flexion caused by the loss of voluntary movement of tibialis anterior controlled by the brain following the injury of the contralateral brain motor centre or conduction path [62].

Research conducted by Zhao et al. [63] reveals that the regulation of form, in addition to the regulations of spirits and breathing in traditional Chinese medicine, requires patients to concentrate on the performance of specific movements. In line with the principle of “skillful use-dependence” to promote the plasticity of the central nervous system, this process brings about greater benefits to the functional reorganization of the central nervous system and the recovery of motor function.

Peng Master's thesis “Clinical Study on Hemiplegia Treatment by Traditional Dao Yin Exercise Combined with Modern Kinesitherapy” [64] suggests that rehabilitation techniques that integrate traditional Chinese medicine with Western medicine produce beneficial effects on the treatment of hemiplegia, especially old hemiplegia, and the technique used in traditional Chinese medicine here is Dao Yin exercise. Peng Yue believes that reasonable practice of Dao Yin exercise based on specific conditions will help to establish a correct exercise model, thereby preventing and curing diseases, enhancing physical fitness, and accelerating rehabilitation. During the recovery of brain injury, Dao Yin exercise may soothe nerves, reduce intracranial pressure, and cure edema in the acute phase, and during the chronic convalescence, it may contribute to the formation of cerebral collateral circulation as well as the reestablishment of foci of excitation in the central nervous system.

These evidences suggest that Dao Yin exercise has remarkable therapeutic effects on central nervous system disorders, especially hemiplegia and related diseases. Practicing Dao Yin exercise can better develop and functionally reorganize the central nervous potential, thereby effectively promoting recovery.

### 6.6. Therapeutic Effects of Dao Yin Exercise on Immune Dysfunction

Immunity refers to the physiological balanced state of an organism having an immune system to tell itself from unwanted biological invasion and to defend against antigenic foreign matters through immune responses. With the increase in age, middle-aged and elderly people are subject to rapid decline in immune function, becoming more likely to be infected by various pathogens and inflicted with tumours and immune diseases. Effective practice of physical exercise keeps biological function in a normal state, enhances cognition, self-care, activity, and other abilities of the elderly, and improves their quality of life, thus delaying the process of aging [65].

The article “Effect of Health Qigong Mawangdui Daoyin on NK Cell of Old and Middle-aged Women” by Wang et al. [4] probes into the effect of Dao Yin exercise on improving immune function of middle-aged and elderly women. The researchers believe that long-term regular practice of Mawangdui Dao Yin exercise can alleviate tension caused by autonomic response, and that the decline of autonomic nervous activity can improve cell-mediated immunoregulation function as well as increase the quantity and viability of immune cells.

Yu and Wang [65] observed the changes of T lymphocytes in the peripheral blood of 100 healthy middle-aged and elderly people before and after their 6-month practice of Wu Qin Xi, a form of health Qi Gong. After 6 months, compared with baseline levels, the male subjects in the experimental group had significantly decreased CD8+ and significantly increased the ratio of CD4+/CD8+ (*P* < 0.05); the male subjects in the control group had significantly decreased CD8+ (*P* < 0.05); the female subjects in the experimental group had significantly increased CD3+ (*P* < 0.05), significantly decreased CD8+, and significantly increased the ratio of CD4+/CD8+ (*P* < 0.01). The research results indicate that the 6-month practice of Wu Qin Xi produced positive effects on T lymphocytes in the peripheral blood of middle-aged and elderly people; therefore, such exercise has a regulatory effect on the immune balance of the middle-aged and elderly.

### 6.7. Therapeutic Effects of Dao Yin Exercise on Psychological Illness

Psychotherapy is a form of Dao Yin exercise requiring thoughtless awareness during the practice, which is actually an active psychological adjustment exercise. Dao Yin exercise attaches importance to mental tranquilization and proper language induction, which allow the exerciser to adjust emotions, smooth the internal paths for *qi* movement, and thus return to the normal state with all annoyances eliminated and all cares forgotten [29]. Furthermore, existing research has demonstrated that traditional Chinese Dao Yin exercise simultaneously improves the physiological and psychological functions of the human body, and that its influences on these two functions are interdependent and mutually restrictive [3].

Li et al. [3] believed that long-term active practice of Dao Yin exercise can help a patient to adjust the mind, relive spiritual tension, improve emotional stability, and reduce psychological stress, thereby maintaining mental health.

Liu et al. [66] selected 80 healthy women as research subjects and conducted a controlled experiment using the Self-Generate Physiological Coherence System (SPCS). The subjects were divided into the control group (those who had no recent exercise habits) and the experimental group (those who had no recent exercise habits but had begun to learn Mawangdui Dao Yin exercise recently), each group consisting of 40 subjects. During the experiment, those in the control group had irregular practice of physical exercise, while those in the experimental group learned and practiced Mawangdui Dao Yin exercise. Their emotional and physiological indicators were analysed through the SPCS, and significant changes were found in the frequency- and time-domain indicators (mean heart rate, mean-square deviation of normal PR interval, and high frequency power) of the experimental group. Thus, it was concluded that the practice of Mawangdui Dao Yin exercise can improve and ease the emotions of middle-aged and elderly women.

Ma et al. [67] randomly divided 80 middle-aged and elderly female subjects into the exercise group and the control group, each consisting of 40 subjects. After the experiment started, the subjects in the exercise group were aggregated and trained in Mawangdui Dao Yin exercise three times a week, one hour each time, for 20 weeks; those in the control group did not participate in any group or individual planned physical exercise. By applying the Profile of Mood States (POMS) Scale [68] and the Self-Rating Anxiety Scale (SAS) [69] questionnaire surveys on the subjects before and after the 20-week experiment, the researchers found significant difference (*P* < 0.01) in the exercise group with regard to the subjects' stress, anger, fatigue, depression, energy, sense of dignity, total scores, average total scores, good-quality average scores, and bad-quality average scores before and after their 20-week exercise. All the indicators and scores of the control group showed no significant difference (*P* > 0.05) before and after the experiment.

Wan [70] researched into the physical appearance, sense of happiness, sense of sadness, and psychological stress of the sophomores at Huaiyin Normal University in Jiangsu Province in a study utilizing the practice of Ba Duan Jin. Through questionnaire surveys before and after their 8-week practice of Ba Duan Jin, Wan found evidence that the practice of Ba Duan Jin played a significant role in the promotion of students' psychological health.

Liu et al. [71] randomly selected 69 patients having type 2 diabetes mellitus (T2DM) with symptoms of depression from a community in Beijing and divided them into two groups. Using the self-rating depression scale (SDS) [72] and the quality-of-life scale for patients with type 2 diabetes mellitus (DMQLS) [73], the two groups of patients were assessed with regard to their symptoms of depression and quality of life before and after their 12-week practice of Ba Duan Jin. Meanwhile, their venous blood was collected to test the level of glycosylated haemoglobin (HbA_1_c). As suggested by the research results, long-term regular practice of Ba Duan Jin can effectively relieve the depression symptoms of T2DM patients and improve their quality of life, especially in the aspects of psychology and sense of satisfaction. The results also indicated the benefit of having kept the blood glucose of patients at a stable level.

Zhong et al. [74] selected 74 women with climacteric syndrome as research subjects, whose physical and mental states were evaluated by the SCL-90 [75] and Kupperman index [76] before and after their 6-month practice of Yi Jin Jing. Based on the comparison of their conditions before and after the 6-month course, it was found that the practice of Yi Jin Jing can effectively relieve the physical and mental symptoms of menopause.

## 7. Conclusions

Dao Yin exercise is one of the regimens dating back to ancient China. Through breathing exercise, the flexing and stretching of the limbs, and the regulations of the body and consciousness (i.e., regulating breathing, regulating the form, and regulating the mind), the practice of Dao Yin exercise leads to health preservation, body strengthening, disease prevention, disease treatment, and the development of potential and longevity. As one of the effective therapies of traditional Chinese medicine, it promotes the metabolism of the human body and facilitates the formation and transformation of essence, *qi*, blood, and body fluid. It also dredges the main and collateral channels, regulates *qi* and blood, balances Yin and Yang, and improves the immunity of the body. Recent basic science research has shed light on the potential mechanisms, and increasing number of clinical trials has provided evidence on the effectiveness of Dao Yin on various disorders. These findings have provided the empirical support for the dissemination of Dao Yin to those who can benefit from these traditional healing practices.
